# ICTV Virus Taxonomy Profile: *Cruliviridae* 2023

**DOI:** 10.1099/jgv.0.001930

**Published:** 2023-12-20

**Authors:** Jens H. Kuhn, Scott Adkins, Katherine Brown, Juan Carlos de la Torre, Michele Digiaro, Holly R. Hughes, Sandra Junglen, Amy J. Lambert, Piet Maes, Marco Marklewitz, Gustavo Palacios, Takahide Sasaya, Massimo Turina, Yong-Zhen Zhang

**Affiliations:** ^1^​ Integrated Research Facility at Fort Detrick, National Institute of Allergy and Infectious Diseases, National Institutes of Health, Fort Detrick, Frederick MD 21702, USA; ^2^​ United States Department of Agriculture, Agricultural Research Service, US Horticultural Research Laboratory, Fort Pierce, FL 34945, USA; ^3^​ Division of Virology, Department of Pathology, Addenbrookes Hospital, University of Cambridge, Cambridge CB2 0QN, UK; ^4^​ Department of Immunology and Microbiology IMM-6, The Scripps Research Institute, La Jolla, CA 92037, USA; ^5^​ CIHEAM, Istituto Agronomico Mediterraneo di Bari, 70010 Valenzano, Italy; ^6^​ Centers for Disease Control and Prevention, Fort Collins, CO 80521, USA; ^7^​ Institute of Virology, Charité-Universitätsmedizin Berlin, Corporate Member of Freie Universität Berlin, Humboldt-Universität zu Berlin, and Berlin Institute of Health, Berlin 10117, Germany; ^8^​ KU Leuven, Rega Institute, Zoonotic Infectious Diseases Unit, 3000 Leuven, Belgium; ^9^​ FIND, 1202 Geneva, Switzerland; ^10^​ Department of Microbiology, Icahn School of Medicine at Mount Sinai, New York, NY 10029, USA; ^11^​ Institute for Plant Protection, National Agriculture and Food Research Organization, Tsukuba, Ibaraki 305-8517, Japan; ^12^​ Institute for Sustainable Plant Protection, National Research Council of Italy (IPSP-CNR), 10135 Torino, Italy; ^13^​ School of Life Sciences and Human Phenome Institute, Fudan University, Shanghai 201052, PR China

**Keywords:** ICTV Report, taxonomy, *Cruliviridae*, lincruvirus

## Abstract

*Cruliviridae* is a family of negative-sense RNA viruses with genomes of 10.8–11.5 kb that have been found in crustaceans. The crulivirid genome consists of three RNA segments with ORFs that encode a nucleoprotein (NP), a glycoprotein (GP), a large (L) protein containing an RNA-directed RNA polymerase (RdRP) domain, and in some family members, a zinc-finger (Z) protein of unknown function. This is a summary of the International Committee on Taxonomy of Viruses (ICTV) Report on the family *Cruliviridae*, which is available at ictv.global/report/cruliviridae.

## Virion

Crulivirids produce enveloped spherical particles [1, 2].

## Genome

**Fig. 1.**
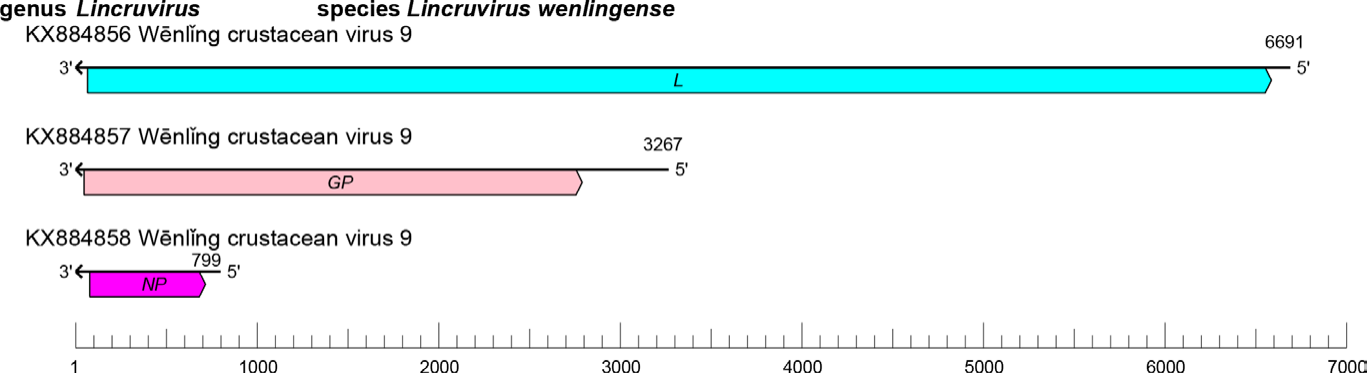
Genome organization of Wēnlǐng crustacean virus 9. ORFs are coloured according to the predicted protein function (*GP*, glycoprotein gene; *L*, large protein gene; *NP*, nucleoprotein gene).

The crulivirid genome comprises three segments (small [S], medium [M] and large [L]) of linear negative-sense RNA with a total length of 10.8–11.5 kb (S segment: about 0.8–1.6 kb; M segment: about 3.2–3.6 kb; and L segment: about 6.7 kb) (Table 1). Each segment contains one ORF that encodes an NP, a GP, and an L protein containing an RdRP domain; some members also encode a Z protein of unknown function on the S segment [1, 3, 4] (Fig. 1).

**Table 1.** Characteristics of members of the family *Cruliviridae.*

Example	Wēnlǐng crustacean virus 9 (S: KX884858; M: KX884857; L: KX884856), species *Lincruvirus wenlingense*, genus *Lincruvirus*
Virion	Enveloped, spherical
Genome	10.8–11.5 kb of trisegmented negative-sense RNA
Replication	Largely undefined
Translation	Unknown
Host range	Portunid crustaceans (crabs)
Taxonomy	Realm *Riboviria*, kingdom *Orthornavirae*, phylum *Negarnaviricota*, class *Ellioviricetes*, order *Bunyavirales*; the family includes the genus *Lincruvirus* and several species

## Replication

Enveloped virions enter host cells via endocytosis; morphogenesis occurs in small and large intracellular vesicles that release mature virions [1, 2].

## Taxonomy

**Fig. 2.**
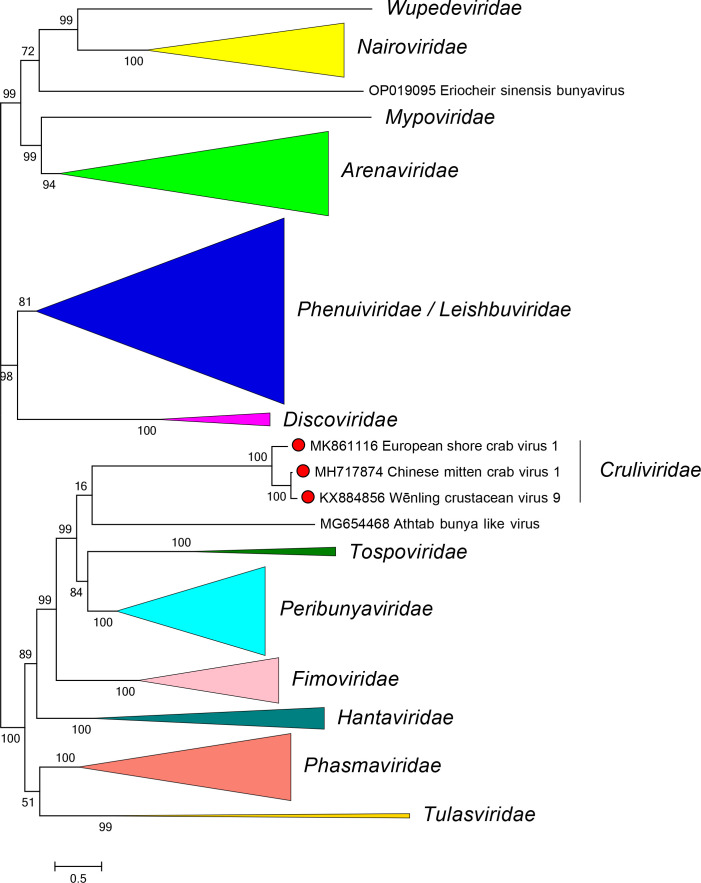
Phylogenetic relationships of viruses in the family *Cruliviridae*. Branches for viruses in other families are collapsed. Numbers at nodes indicate bootstrap support where this was >70 %. Details of the virus sequences and methods used are available in the full ICTV Report on the family *Cruliviridae*.

Current taxonomy: ictv.global/taxonomy. Crulivirids are most closely related to fimovirids, hantavirids, peribunyavirids, phasmavirids, tospovirids and tulasvirids [4–6] (Fig. 2). The family includes the genus *Lincruvirus* and several species for viruses that infect crustaceans. Crulivirids (i) have multisegmented, negative-sense single-stranded RNA genomes; (ii) encode proteins with high sequence identity to proteins of other bunyavirals; (iii) and have five conserved motifs (A–E) in their RdRP domain.

## Resources

Full ICTV Report on the family *Cruliviridae*: ictv.global/report/cruliviridae.

## References

[1] Bojko J, Subramaniam K, Waltzek TB, Stentiford GD, Behringer DC (2019). Genomic and developmental characterisation of a novel bunyavirus infecting the crustacean *Carcinus maenas*. Sci Rep.

[2] Bojko J, Stebbing PD, Dunn AM, Bateman KS, Clark F (2018). Green crab *Carcinus maenas* symbiont profiles along a North Atlantic invasion route. Dis Aquat Organ.

[3] Shi M, Lin X-D, Tian J-H, Chen L-J, Chen X (2016). Redefining the invertebrate RNA virosphere. Nature.

[4] Huang P, Zhang X, Ame KH, Shui Y, Xu Z (2019). Genomic and phylogenetic characterization of a bunya-like virus from the freshwater Chinese mitten crab *Eriocheir sinensis*. Acta Virol.

[5] Olendraite I, Brown K, Firth AE (2023). Identification of RNA virus-derived RdRp sequences in publicly available transcriptomic data sets. Mol Biol Evol.

[6] Herath V, Romay G, Urrutia CD, Verchot J (2020). Family level phylogenies reveal relationships of plant viruses within the order *Bunyavirales*. Viruses.

